# Age- and sex-specific effects on weight loss outcomes in a comparison of sleeve gastrectomy and Roux-en-Y gastric bypass: a retrospective cohort study

**DOI:** 10.1186/2052-9538-1-12

**Published:** 2014-08-11

**Authors:** Sean Manning, Nicholas C Carter, Andrea Pucci, Alexander Jones, Mohamed Elkalaawy, Wui-hang Cheung, Borzoueh Mohammadi, Nicholas Finer, Alberic G Fiennes, Majid Hashemi, Andrew D Jenkinson, Marco Adamo, Rachel L Batterham

**Affiliations:** Centre for Obesity Research, Rayne Institute, Department of Medicine, University College London, Rayne Building, 5 University Street, London, WC1E 6JJ UK; UCLH Centre for Weight Loss, Metabolic and Endocrine Surgery, University College London Hospitals, Ground Floor West Wing, 250 Euston Road, London, NW1 2PG UK; National Institute of Health Research University College London Hospitals Biomedical Research Centre, London, W1T 7DN UK; Queen Alexandra Hospital, Southwick Hill Road, Portsmouth, PO6 3LY UK; University College London Institute of Cardiovascular Science, 170 Tottenham Court Road, London, W1T 7HA UK; Clinical and Experimental Surgery Department, Medical Research Institute, University of Alexandria, Hadara, Alexandria, 21561 Egypt; Surrey Weight Loss Centre, St Anthony’s Hospital, North Cheam, SM3 9DW UK

**Keywords:** Obesity, Bariatric surgery, Gastric bypass, Sleeve gastrectomy, Weight loss, Diabetes, BMI

## Abstract

**Background:**

Roux-en-Y gastric bypass (RYGBP) and sleeve gastrectomy (SG) are the most common bariatric procedures undertaken globally but there are no evidenced-based criteria that inform the selection of one operation over the other. The purpose of this study was thus to compare weight loss outcomes between RYGBP and SG, and to define patient factors affecting weight loss.

**Methods:**

A single-centre two-year follow-up retrospective cohort study of all adults who underwent either RYGBP (n = 422) or SG (n = 432) between 2007 and 2012, at University College London Hospitals National Health Service Foundation Trust, an academic tertiary referral centre, was undertaken. Multilevel linear regression was used to compare weight loss between groups, enabling adjustment for preoperative BMI (body mass index) and evaluation for interaction factors.

**Results:**

One- and two-year results showed that unadjusted BMI loss was similar between groups; 13.7 kg/m^2^ (95% CI: 12.9, 14.6 kg/m^2^) and 12.8 kg/m^2^ (95% CI: 11.8, 13.9 kg/m^2^) for RYGBP patients respectively compared with 13.3 kg/m^2^ (95% CI: 12.0, 14.6 kg/m^2^) and 11.5 kg/m^2^ (95% CI: 10.1, 13.0 kg/m^2^) for SG patients respectively. Adjusting for preoperative BMI, there was 2.2 kg/m^2^ (95% CI: 1.5, 2.8) and 2.3 kg/m^2^ (95% CI: 1.3, 3.3) greater BMI loss in the RYGBP group compared to the SG group at one and two years respectively (*P* < 0.001 for both). The interaction analyses demonstrated that age and sex had important differential impacts on SG and RYGBP weight outcomes. Men under 40 and women over 50 years obtained on average far less benefit from SG compared to RYGBP, whereas men over 40 years and women under 50 years experienced similar weight loss with either procedure (*P* = 0.001 and 0.022 for interaction effects at one and two years respectively).

**Conclusions:**

Our results show that patient sex and age significantly impact on weight loss in a procedure-dependent manner and should be considered when choosing between RYGBP and SG. Optimizing procedure selection could enhance the effectiveness of bariatric surgery, thus further increasing the benefit-to-risk ratio of this highly effective intervention.

**Electronic supplementary material:**

The online version of this article (doi:10.1186/2052-9538-1-12) contains supplementary material, which is available to authorized users.

## Background

The prevalence of severe obesity is rising rapidly [1]. Moreover, the disproportionately high health and socioeconomic burden associated with severe obesity [2] emphasises the need not only for effective prevention strategies but also effective interventions for patients seeking obesity treatment. Bariatric or metabolic surgery is currently the most effective weight loss intervention for the severely obese, is cost-effective and results in significant reductions in morbidity and mortality [3, 4]. Roux-en-Y gastric bypass (RYGBP) is considered the ‘gold standard’ bariatric procedure, with robust long-term clinical outcomes [3, 5], and until recently had been unrivalled in terms of benefit-to-risk ratio [6]. Recently, there has been a notable shift in the types of bariatric procedures being performed, with a dramatic increase in the proportion of sleeve gastrectomy (SG) procedures from 5% of all performed globally in 2008 to 28% in 2011 [7]. The increase in SG operation number reflects the recent implementation of SG as a ‘stand-alone’ procedure [8], and was accompanied by a proportionate decline in the number of adjustable gastric band (AGB) procedures (42% in 2008 to 18% in 2011), which now places SG as the second most common procedure after RYGBP (47% in 2011) [7]. Accordingly, the effectiveness of SG compared with RYGBP, has come under increasing scrutiny. Multi-centre studies in the United States (US) reported that the weight loss and safety outcomes of SG are positioned between RYGBP and AGB [9, 10]. On the other hand, randomised controlled trials comparing SG with RYGBP showed similar efficacy, albeit with outcome data limited to one to three years to date [11–16].

Previous studies have identified clinical factors that are associated with adverse weight loss outcomes post-bariatric surgery, although primarily for RYGBP. For example, biological factors such as higher baseline BMI, older age, male sex and type 2 diabetes (T2D) are consistent predictors of less beneficial results [17–23]. However, relatively few studies have examined the effects of such clinical characteristics factors in relation to SG [23]. Furthermore, the mere presence or absence of these biological factors is likely to have a limited impact on the decision to proceed with bariatric surgery for patients who are fit for surgery. We argue that identification of factors that have differential effects on SG and RYGBP outcomes is a more constructive approach, as this could influence decision-making when choosing between RYGBP and SG. Two previous studies have explored whether age could influence weight loss differentially according to procedure, one of which had a much smaller sample size than in our study [18], and the other compared RYGBP and AGB only [22]. Thus, in relation to SG and RYGBP, there is insufficient evidence to allow individualised recommendation of one procedure over the other [24]. In addition, clinical and translational research has shown that the biological mechanisms governing the effects of RYGBP and SG are different [25–30].

We thus hypothesized that age, sex and/or T2D status could have different effects on weight loss outcomes for each procedure. In this regard, we anticipated one of three possible scenarios: (1) that these patient factors, alone or in combination, could negatively affect the weight loss outcome in a comparable manner for both procedures (i.e. no interaction effect) (2) that these patient factors, alone or in combination, could negatively affect the weight loss outcome of one procedure but not the other (i.e. interaction effect), (3) that these patient factors, alone or in combination, could negatively affect the weight loss outcome for both procedures but to varying degrees (i.e. interaction effect). The exploratory aim of the study was to define clinical characteristics affecting weight loss, in order to optimize the recommendation of appropriate procedure for patients undergoing bariatric surgery. This study, with two-year postoperative follow-up data, represents the largest single-centre comparison of RYGBP and SG weight loss outcomes.

## Methods

### Study design

This study was designed as a retrospective cohort study and was undertaken at University College London Hospitals NHS Foundation Trust. Data were obtained by review of prospectively-maintained electronic clinical data records and clinical casenotes within a single bariatric surgery unit in an academic tertiary referral centre.

### Study participants and setting

All patients aged 18 or over, with a BMI ≥ 40.0 kg/m^2^, or ≥ 35.0 kg/m^2^ in the presence of at least one obesity-related comorbidity, who underwent either RYGBP or SG as a primary bariatric procedure were included. All patients fulfilled National Institute of Clinical Excellence criteria for bariatric surgery [31]. Patients were evaluated pre- and postoperatively by a multidisciplinary team (MDT) consisting of surgeons, physicians, clinical nurse specialists, psychologists, dietitians, and anesthetists. All patients were provided with both written and verbal information detailing each bariatric procedure, including the risks and benefits, prior to giving their written informed consent. The study protocol was approved by the National Health Service Research Ethics Committee (ID#09/H0715/65) and was in compliance with the Helsinki Declaration.

### Pre- and postoperative protocols

The practice in our centre is to offer either SG or RYGBP to all candidates deemed suitable for bariatric surgery. The decision for procedure selection is based on informed patient preference after standardised counselling including details, risks and benefits of each procedure. While there are no absolute BMI restrictions for RYGBP, the practice in our centre is to advise patients with a BMI ≥60.0 kg/m^2^ that SG is a more appropriate procedure due to technical considerations. Patients were advised to follow a two-week preoperative low energy diet, with the aim of reducing liver size. Regarding postoperative T2D management, the standard practice in our centre has been to stop glucose-lowering medications pre-discharge if glucose levels remain satisfactorily controlled off therapy. Postoperatively, patients were advised to follow a liquid diet for two weeks, followed by softer foods for two weeks, before moving onto more textured foods for the next two weeks and resuming a solid diet thereafter. Patients were subsequently reviewed in accordance with a predefined postoperative follow-up plan; telephone follow-up within a week of discharge from the specialist nurse, postoperative hospital clinic review at 6 weeks by the specialist nurse, at 3 months by the dietitian and surgeon, at 6 months by the surgeon and 6- to 12-monthly by the surgeon thereafter. Weight was measured at each hospital clinic visit, by a trained healthcare assistant, using a Walkthrough Platform A12SS Stainless Steel Indicator, and height was measured using a wall-mounted digital stadiometer.

### Surgical technique- RYGBP

RYGBP was performed using a laparoscopic, antecolic, antegastric RYGBP. A 30-40 ml gastric pouch was fashioned. The alimentary limb was measured at 120 cm. The omentum was divided longitudinally and a stapled jejunojejunal anastomosis was performed. The gastric pouch was created with 1.8 mm staple height cartridges. A linear stapler technique (creating a 1.2 cm stoma with suturing of the enterotomies), a circular stapler technique (deploying the 21 mm anvil into the gastric pouch through a service gastrotomy) or an entirely sutured gastrojejunostomy (calibrated over a 32-French bougie to give a 1.2 cm stoma) were used for the gastrojejunal anastomosis.

### Surgical technique- SG

SG was performed using a standard laparoscopic technique. Five-port access was created including one port for liver retraction. The greater curvature of stomach was mobilised (3-5 cm) from the pylorus to the angle of His. A 32-French bougie was passed by the anaesthetist to lie in the oesophagus and through to the pylorus. The sleeve was created around the bougie using a laparoscopic stapler, 2.0 mm staple height on the gastric antrum and body and 1.8 mm staple height for the rest of the stomach, with staple line reinforcement.

### Outcomes

Postoperative weight loss, expressed as BMI loss, was determined relative to the weight on the day of operation. Postoperative weights were estimated at standard postoperative timepoints (monthly for the first three months and three-monthly thereafter), using linear interpolation between the two nearest actual measurement times either side of the standard timepoint. Available postoperative weight data were excluded only if SG was converted to RYGBP or RYGBP was reversed, and postoperative weight data until the point of the second procedure were still included in these circumstances. Complete or partial T2D remission was defined using American Diabetes Association consensus group criteria (HbA1c <6.5%/48 mmol/mol beyond one year and no active pharmacologic therapy) [32]. Complications were graded using the Clavien-Dindo classification of surgical complications [33].

### Statistical analysis

In order to adjust for preoperative BMI in comparing weight outcomes, we performed repeated-measures multilevel multiple linear regression analyses. Differences in BMI loss between procedures and interactions with sex, age and T2D were assessed by estimating marginal means for the different sub-groups. In a sub-group analysis of patients with T2D, logistic regression with adjustment for baseline factors was used to assess odds of complete or partial T2D remission. Interpolation and analyses were performed with Stata™ software version 13 (StataCorp, Texas, US).

## Results

**Figure 1:**
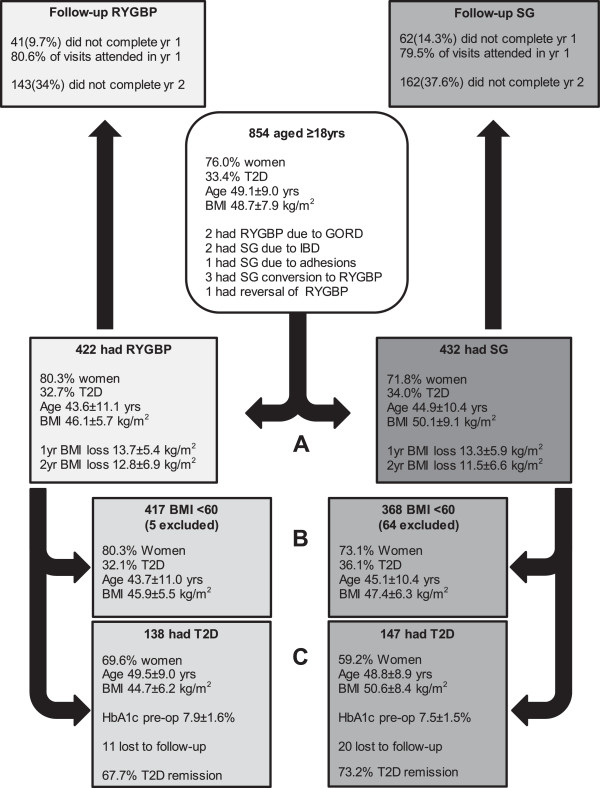
**Analyses included (A) the entire cohort of patients who had RYGBP or SG as a primary procedure (Additional file** 1 **: Table S1), (B) the sub-group of patients with baseline BMI < 60 kg/m2 (Additional file** 1 **: Table S4) and (C) the sub-group of patients with T2D (Additional file** 1 **: Table S6).** All available postoperative weight data were included in the analyses, apart from weight data subsequent to the three conversions from SG to RYGBP and the single reversal of RYGBP. Postoperative weight data from patients whose operation was selected due to the presence of severe gastrooesophageal reflux disease (GORD), inflammatory bowel disease (IBD) or history of adhesions were included in the analyses. Full clinical characteristics for patients who did not complete more than one year of follow-up are presented in Additional file 1: Table S8. Age, baseline BMI and preoperative HbA1c are presented in the diagram as mean ± SD. BMI loss, at one and two years, are presented in the diagram as unadjusted results (mean ± SD). T2D remission was defined by ADA consensus group criteria.

A total of 854 adults (Figure 1, Additional file 1: Table S1) underwent either SG (n = 432) or RYGBP (n = 422). There were significant group differences in sex distribution (higher proportion of women in the RYGBP group), baseline BMI (higher in SG group) and preoperative assessment time (longer in SG group), but no significant group differences in age, ethnicity or diabetes status (Additional file 1: Table S1). There were a total of 34 complications associated with RYGBP and 23 with SG (Additional file 1: Table S2). The frequency of Clavien-Dindo-classified complications did not differ between procedures (P = 0.26, chi-squared for trend). One- and two-year results showed that unadjusted BMI loss was similar between groups; 13.7 kg/m^2^ (95% CI: 12.9, 14.6 kg/m^2^) and 12.8 kg/m^2^ (95% CI: 11.8, 13.9 kg/m^2^) for RYGBP patients respectively compared with 13.3 kg/m^2^ (95% CI: 12.0, 14.6 kg/m^2^) and 11.5 kg/m^2^ (95% CI: 10.1, 13.0 kg/m^2^) for SG patients respectively. Using the multilevel model, there was 2.2 kg/m^2^ (95% CI: 1.5, 2.8) and 2.3 kg/m^2^ (95% CI:1.3, 3.3) greater BMI loss in the RYGBP group compared to the SG group at one and two years respectively (P < 0.001 for both).

**Figure 2:**
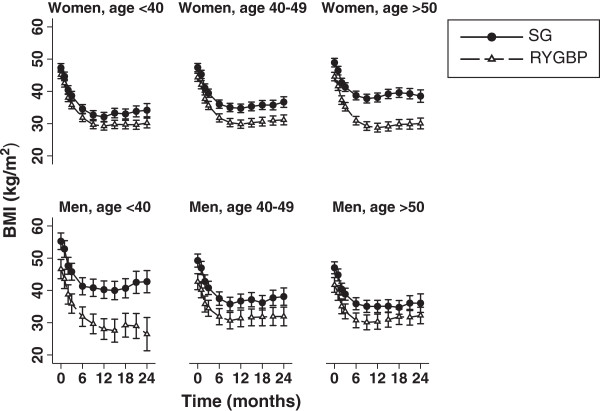
**Estimated marginal mean BMI trajectories, with 95% CI, over a two-year postoperative period for women and men, by age categories (<40 years, 40 to 49 years, ≥50 years) in SG and RYGBP groups.**

Time by procedure interaction analyses were undertaken to assess the influence of patient factors (sex, age and diabetes status) on BMI loss between groups. We found:

(1) a significant time × procedure × sex interaction; RYGBP and SG led to comparable weight loss in men, but women in the RYGBP group had greater weight loss than those in the SG group. Thus, the differences in BMI loss between procedures in women (RYGBP > SG) were 2.6 kg/m^2^ (95% CI: 1.1, 4.1, P = 0.001) and 3.1 kg/m^2^ (95% CI: 0.8, 5.3, P = 0.008) greater than the differences between procedures in men (RYGBP ≈ SG), at one and two years respectively.

(2) a significant time × procedure × age interaction, manifesting in less favourable outcomes in the SG group with increasing age; for six- (β = 0.08, P = 0.012), nine- (β = 0.08, 0.012) and twelve-month (β = 0.07, 0.018) postoperative timepoints.

(3) a significant time × procedure × sex × age interaction. On this occasion, the interaction was directed towards more favourable outcome in the SG group with increasing age for men, with significant effects at nine-month (β = -0.19, P = 0.012, twelve-month (β = -0.26 P = 0.001), eighteen-month (β = -0.26, P = 0.014) and two-year (β = -0.31, P = 0.022) postoperative timepoints. In fact, using age categorisation (<40, 40–49, ≥50 years) (Additional file 1: Table S3), this interaction was found to represent two distinct phenomena (Figure  2). Firstly, for men, the difference in BMI loss between procedures was most pronounced in men <40 (RYGBP > SG) and progressively diminished with increasing age category. Secondly, the opposite trend was observed for women; BMI loss between procedures was similar in women <40 and a divergence in BMI loss was visualised with increasing age category (RYGBP > SG) (Figure  2).

(4) no significant interaction of T2D status or preoperative glycaemic control with procedure.

Results of the multilevel models were not meaningfully altered (Additional file 2: Figure S1) upon exclusion of data from patients with a baseline BMI ≥60.0 kg/m^2^ (Additional file 1: Table S4) from the analyses or by adjusting for preoperative weight loss (Additional file 1: Table S1 and Additional file 1: Table S5).

In order to determine whether sex and age also impact upon T2D remission in a procedure-specific manner, a sub-group analysis of patients with T2D was undertaken (Additional file 1: Table S6). Adjusting for preoperative BMI, glycaemic control and intensity of therapeutic regimen, procedure-dependent sex and age effects were identified. Sex predicted T2D remission in the RYGBP (OR = 0.37, 95% CI: 0.14, 0.95, P = 0.039) but not the SG group (OR = 1.14, 95% CI: 0.36, 3.65, P = 0.83) (Additional file 1: Table S7). Increasing age was associated with a reduced odds of diabetes remission in the SG (OR = 0.35, 95% CI: 0.14, 0.91, P = 0.032) but not the RYGBP group (OR = 1.27, 95% CI: 0.67, 2.40, P = 0.47). Upon inclusion of one-year BMI loss in the model, the significant associations with T2D remission, both for sex (P = 0.11) and age (P = 0.25), were lost.

## Discussion

Our findings, from the largest single-centre study comparing the effectiveness of SG and RYGBP, confirm SG as a valid alternative to RYGBP for most patients. However, we have identified clinical characteristics that could influence procedure selection between RYGBP and SG for patients undergoing bariatric surgery. In terms of weight loss, men < 40 and women ≥ 50 years obtained on average less benefit from SG compared to RYGBP. In contrast, men ≥ 40 years and women < 50 years experienced similar weight loss with either procedure. Furthermore, the results of the sub-group analysis of patients with T2D supported this concept of differential effects of age and sex on procedure outcome. In terms of T2D remission, relative to women, men fared comparatively better with SG than with RYGBP, which was consistent with the sex interaction results for weight change. In addition, there were less favourable T2D remission outcomes, with increasing age, in the SG but not the RYGBP group, again consistent with the age interaction results for weight change. Interestingly, these respective associations with T2D remission were lost upon inclusion of one-year BMI loss in the model, suggesting that there are common or overlapping mechanisms underlying the age- and sex-specific effects on weight loss and T2D amelioration. Taken together, these findings provide a strong basis for a prospective randomized study, which could definitively address whether individualization of procedure selection, using clinical factors such as age and sex, could achieve better outcomes than standard care.

There are a number of biologically plausible explanations for the striking differential effects of age and sex on procedure-specific outcome. Since SG and RYGBP are characterised by distinct anatomical modifications to the gastrointestinal (GI) tract, unsurprisingly, there are procedure-specific alterations to gut hormones profiles [28, 30], which in turn could potentially underlie the sex- and age-dependent differences between procedures detected in our study. For example, levels of ghrelin, an orexigenic gut hormone primarily secreted by X/A cells in the fundus and body of the stomach, fall predictably after SG [28, 30, 34], and remain consistently low at one year [30, 34], in contrast with post-RYGBP ghrelin levels which return to baseline by or before one year [34]. Our findings are consistent with a previous study that examined whether age-specific differences in weight loss were present after RYGBP or AGB [22]. This study, which did not include SG patients, found that compared to women aged 20–45, women aged 55–65 had achieved significantly less weight loss after AGB, a purely restrictive procedure, but not after RYGBP, and that no such relationship existed in men. The authors postulated that menopausal status could potentially affect bariatric surgery outcome in a procedure-dependent manner. Translational research using animal models suggests that absence of oestrogen alters ghrelin responses [35], which could potentially explain the altered effects of SG in women who are over the age of 50. Interestingly, in men, weight outcomes with SG improved relative to RYGBP with increasing age, suggesting that a sex-specific factor is important for SG response.

There are important limitations of this retrospective cohort study. First is the potential selection bias that is inherent in any non-randomized study. However, informed patient preference was the overriding influence on procedure selection in our centre. Since all patients received the same preoperative counselling, procedure selection occurred in a pseudo-random fashion for the vast majority of patients, evidenced by the near equivalent numbers of patients undergoing SG and RYGBP. Furthermore, we accounted for preoperative BMI by using a multilevel linear model and the results were not altered meaningfully upon excluding from the analysis the most likely source of bias (data from patients with a baseline BMI ≥60.0 kg/m^2^). Nevertheless, the results should be interpreted with caution due to the possibility of an unmeasured bias in the procedure selection. Secondly, there was considerable postoperative attrition, a consistent feature of bariatric surgery cohort studies [9]. Although follow-up between SG and RYGBP groups (Figure 1) and baseline clinical characteristics of non-completers compared to completers were very similar in our study (Additional file 1: Table S8), the absence of complete follow-up for approximately one-third of patients further necessitates that the results be interpreted with caution. Thirdly, long-term weight data were not available for this study, therefore whether the findings observed persist beyond two years is unknown. Finally, the subgroup analyses used in our study may have limited the power to detect important effects. For example, the lack of an effect on BMI loss observed in the T2D by procedure interaction analyses could be explained by the smaller sample size of patients with T2D in the cohort.

## Conclusions

In summary, in the largest single-centre comparison of RYGBP and SG outcomes, we report robust weight loss for both procedures, with few major complications. Our results demonstrate that patient sex and age significantly impact on weight loss and diabetes remission in a procedure-dependent manner. These findings provide a strong basis for a further prospective randomized study, which could definitively address whether individualization of procedure selection, using age- and sex-specific criteria, could achieve better outcomes than standard care. Optimizing procedure selection could enhance the effectiveness of bariatric surgery, thus further increasing the benefit-to-risk ratio of this highly effective intervention. Investigation of the mechanisms underlying age- and sex-specific aspects of the differential procedure responses is warranted.

## Electronic supplementary material

Additional file 1: Tables S1-8: Baseline demographic, anthropometric and clinical characteristics of entire cohort and subgroups analysed. (DOCX 53 KB)

Additional file 2: Figure S1: Estimated marginal mean BMI trajectories (±SE) over a two-year postoperative period for women and men, by age categories (<40 years, 40 to 49 years, ≥50 years) in SG and RYGBP groups, excluding data from patients with a baseline BMI ≥60.0 kg/m^2^. (PDF 41 KB)
