# Supplementary material for: Age- and sex-specific effects on weight loss outcomes in a comparison of sleeve gastrectomy and Roux-en-Y gastric bypass: a retrospective cohort study
Source: BMC Obes. 2014 Aug 11;1:12. doi: 10.1186/2052-9538-1-12 (PMC4510900; doi:10.1186/2052-9538-1-12)
Supplement: Supplementary file 1 — Additional file 1: Tables S1-8: Baseline demographic, anthropometric and clinical characteristics of entire cohort and subgroups analysed. (DOCX 53 KB) [file 40608_2014_12_MOESM1_ESM.docx]

**Supplementary Data**

**Supplementary Table 1. Baseline demographic, clinical and anthropometric characteristics of entire cohort**

|  |  | Total | RYGBP | SG | *P^*^* |
| --- | --- | --- | --- | --- | --- |
|  |  | **n, %** | **n, %** | **n, %** |  |
|  |  | 854 (100) | 422 (49.4) | 432 (50.6) |  |
|  |  |  |  |  |  |
| Women |  | 649 (76.0) | 339 (80.3) | 310 (71.8) | 0.003 |
| Men |  | 205 (24.0) | 83 (19.7) | 122 (28.2) |  |
|  |  |  |  |  |  |
| Ethnicity; white |  | 669 (78.5) | 332 (79.0) | 337 (78.0) | 0.71 |
| other |  | 185 (21.5) | 90 (21.0) | 95 (22.0) |  |
|  |  |  |  |  |  |
| T2D |  | 285 (33.4) | 138 (32.7) | 147 (34.0) | 0.44 |
|  |  |  |  |  |  |
| Hypertension |  | 333 (40.0) | 155 (36.7) | 178 (41.2) | 0.18 |
|  |  |  |  |  |  |
| Statin therapy |  | 225 (26.3) | 113 (26.8) | 112 (25.9) | 0.78 |
|  |  |  |  |  |  |
| Obstructive sleep apnoea |  | 148 (17.3) | 63 (14.9) | 85 (19.7) | 0.067 |
|  |  |  |  |  |  |
| Depression |  | 226 (26.5) | 121 (28.7) | 105 (24.3) | 0.15 |
|  |  |  |  |  |  |
| Vascular disease^1^ |  | 29 (3.4) | 12 (2.9) | 17 (3.9) | 0.38 |
|  |  |  |  |  |  |
| Chronic kidney disease^2^ |  | 23 (2.7) | 11 (2.6) | 12 (2.8) | 0.37 |
|  |  |  |  |  |  |
| Previous cancer |  | 15 (1.7) | 6 (1.4) | 9 (2.1) | 0.46 |
|  |  |  |  |  |  |
|  |  | **mean (SD)** | **mean (SD)** | **mean (SD)** |  |
| Age (yrs) | | 44.3 (10.7) | 43.6 (11.1) | 44.9 (10.4) | 0.09 |
|  | |  |  |  |  |
| BMI (kg/m^2^) | | 48.1 (7.9) | 46.1 (5.7) | 50.1 (9.1) | <0.0001 |
|  | |  |  |  |  |
| Pre-op assessment^3^ (mths) | | 8.4 (5.8) | 7.9 (5.8) | 8.9 (5.7) | 0.01 |
|  | |  |  |  |  |
| Pre-op weight loss (kg) | | 4.8 (7.2) | 4.6 (6.6) | 5.0 (7.8) | 0.35 |
|  | |  |  |  |  |

*Sex distribution, ethnicity distribution and prevalence of comorbidities were compared between procedures using Pearson’s chi-squared tests. Comparisons of means between procedures were performed using an unpaired two-tailed t-tests.

**^1^**Combination of history of myocardial infarction, ischaemic heart disease, coronary artery bypass grafting, stroke, transient ischaemic attack and peripheral vascular disease

^2^Chronic kidney disease (CKD) stage 3-5

^3^Duration of preoperative assessment period

**Supplementary Table 2. Summary of complications according to Clavien-Dindo classification**

| **Grade** | **RYGBP** | **SG** |
| --- | --- | --- |
| 1 | 1 | 1 |
| 2 | 10 | 7 |
| 3a | 14 | 5 |
| 3b | 9 | 8 |
| 4a | 0 | 0 |
| 4b | 0 | 1^a^ |
| 5 | 0 | 1^b^ |
| Total | 34 | 23 |

^a^ multi-organ dysfunction requiring a prolonged intensive care unit admission subsequent to a gastric leak

^b^ peri-operative due to multi-organ failure subsequent to a gastric leak

**Supplementary Table 3. Summary of numbers per age categories by sex and procedure**

|  | **Age Category (years)** | | |  |
| --- | --- | --- | --- | --- |
|  | **<40** | **40-49** | **≥50** | ***P**** |
| **Women, RYGBP** | 123 | 113 | 103 | 0.6 |
| **Women, SG** | 100 | 109 | 101 |  |
|  |  |  |  |  |
| **Men, RYGBP** | 20 | 29 | 34 | 0.9 |
| **Men, SG** | 27 | 43 | 52 |  |

*The proportions of women and men across the selected age categories were compared between procedures using chi-squared tests for trend.

**Supplementary Table 4. Baseline demographic, clinical and anthropometric characteristics of patients with a baseline BMI <60kg/m^2^**

|  |  | Total | RYGBP | SG | *P** |
| --- | --- | --- | --- | --- | --- |
|  |  | **n, %** | **n, %** | **n, %** |  |
|  |  | 785 (100) | 417 (53.1) | 368 (46.9) |  |
|  |  |  |  |  |  |
| Women |  | 604 (76.9) | 335 (80.3) | 269 (73.1) | 0.016 |
| Men |  | 181 (23.1) | 82 (19.7) | 99 (26.9) |  |
|  |  |  |  |  |  |
| Ethnicity; white |  | 608 (77.4) | 328 (78.7) | 280 (76.1) | 0.32 |
| other |  | 177 (22.6) | 89 (21.3) | 88 (23.9) |  |
|  |  |  |  |  |  |
| Type 2 diabetes |  | 267 (34.0) | 134 (32.1) | 133 (36.1) | 0.24 |
|  |  |  |  |  |  |
| Hypertension |  | 306 (39.0) | 154 (37.0) | 152 (41.3) | 0.24 |
|  |  |  |  |  |  |
| Statin therapy |  | 207 (26.4) | 113 (27.1) | 94 (25.5) | 0.62 |
|  |  |  |  |  |  |
| Obstructive sleep apnoea |  | 131 (16.7) | 62 (14.9) | 69 (18.7) | 0.15 |
|  |  |  |  |  |  |
| Depression |  | 211 (27.0) | 120 (28.8) | 91 (24.7) | 0.20 |
|  |  |  |  |  |  |
| Vascular disease^1^ |  | 28 (3.6) | 12 (2.9) | 16 (4.3) | 0.27 |
|  |  |  |  |  |  |
| Chronic kidney disease^2^ |  | 22 (2.8) | 11(2.6) | 11(3.0) | 0.36 |
|  |  |  |  |  |  |
| Previous cancer |  | 15 (1.9) | 6 (1.4) | 9 (2.4) | 0.30 |
|  |  |  |  |  |  |
|  |  | **mean (SD)** | **mean (SD)** | **mean (SD)** |  |
| Age (yrs) | | 44.3 (10.7) | 43.7 (11.0) | 45.1 (10.4) | 0.061 |
|  | |  |  |  |  |
| BMI (kg/m^2^) | | 46.6 (5.9) | 45.9 (5.5) | 47.4 (6.3) | 0.0004 |
|  | |  |  |  |  |
| Pre-op assessment^3^ (mths) | | 8.2 (5.4) | 7.9 (5.6) | 8.6 (5.3) | 0.10 |
|  | |  |  |  |  |
| Pre-op weight loss (kg) | | 4.2 (6.5) | 4.4 (6.3) | 4.0 (6.8) | 0.43 |
|  | |  |  |  |  |

*Sex distribution, ethnicity distribution and prevalence of comorbidities were compared between procedures using Pearson’s chi-squared tests. Comparisons of means between procedures were performed using unpaired two-tailed t-tests.

**^1^**Combination of history of myocardial infarction, ischaemic heart disease, coronary artery bypass grafting, stroke, transient ischaemic attack and peripheral vascular disease

^2^Chronic kidney disease (CKD) stage 3-5

^3^Duration of preoperative assessment period

| Age category (yrs) | <40 | 40-49 | ≥50 |
| --- | --- | --- | --- |
| Women, RYGBP | 4.1 (±5.2) | 5.1 (±7.3) | 3.8 (±6.2) |
| Women, SG | 2.9 (±7.8) | 4.9 (±7.1) | 4.4 (±6.5) |
| *P** | 0.2 | 0.8 | 0.5 |
|  |  |  |  |
| Men, RYGBP | 3.1 (±7.3) | 7.5 (±7.1) | 4.9 (±6.0) |
| Men, SG | 7.9 (±9.2) | 7.7 (±8.9) | 7.0 (±8.7) |
| *P** | 0.07 | 0.9 | 0.2 |

**Supplementary Table 5. Summary of mean (**±**SD) preoperative weight loss (kg) in each age category by sex and procedure**

*Preoperative weight loss for women and for men, in each age category,

were compared between procedures using unpaired two-tailed t-tests.

**Supplementary Table 6. Clinical characteristics of patients with T2D**

|  | Total | RYGBP | SG | *P** |
| --- | --- | --- | --- | --- |
|  | **n, %** | **n, %** | **n, %** |  |
| Total | 285 (100) | 138 (100) | 147 (100) |  |
| Women | 183 (64.2) | 96 (69.6) | 87 (59.2) | 0.07 |
|  |  |  |  |  |
|  | **mean (SD)** | **mean (SD)** | **mean (SD)** |  |
| Age (yrs) | 49.1 (9.0) | 49.5 (9.0) | 48.8 (8.9) | 0.46 |
| BMI (kg/m^2^) | 48.7 (7.9) | 44.7 (6.2) | 50.6 (8.4) | <0.0001 |
|  |  |  |  |  |
| T2D duration (yrs) | 4.7 (5.3) | 4.8 (4.9) | 4.5 (5.7) | 0.66 |
| HbA1c pre-op (%) | 7.7 (1.6) | 7.9 (1.6) | 7.5 (1.5) | 0.015 |
| HbA1c post-op^1^ (%) | 6.2 (1.1) | 6.1 (1.1) | 6.2 (1.1) | - |
|  |  |  |  |  |
|  | **n, %** | **n, %** | **n, %** |  |
| Preoperative |  |  |  |  |
| HbA1c <7% | 129 (45.3) | 52 (37.7) | 77 (52.4) | 0.06 |
| HbA1c 7-8.4% | 92 (32.3) | 51 (37.0) | 41 (27.9) |  |
| HbA1c ≥8.5% | 64 (22.5) | 35 (25.4) | 29 (19.7) |  |
|  |  |  |  |  |
| Postoperative |  |  |  |  |
| Total | 254 (89.1) | 127 (92.0) | 127 (86.4) |  |
| Lost to follow-up | 31 (10.9) | 11 (8.0) | 20 (13.6) |  |
| HbA1c <7% | 206 (81.1) | 107 (84.3) | 99 (77.9) |  |
| HbA1c 7-8.4% | 36 (14.2) | 15 (11.8) | 21 (16.6) | 0.42 |
| HbA1c ≥8.5% | 12 (4.7) | 5 (3.9) | 7 (5.5) |  |
| Preoperative Medications |  |  |  |  |
| Metformin monotherapy | 92 (32.3) | 46 (33.3) | 46 (31.3) | 0.11 |
| ≥2 OHA^2^ | 55 (19.3) | 34 (24.6) | 21(14.3) |  |
| Insulin | 47 (16.5) | 25 (18.1) | 22 (15.0) |  |
| GLP-1R agonist | 19 (6.7) | 8 (5.8) | 11(7.5) |  |
|  |  |  |  |  |
| Postoperative Medications |  |  |  |  |
| Metformin monotherapy | 21(8.3) | 11 (8.7) | 10 (7.9) | 0.81 |
| ≥2 OHA^3^ | 11 (4.3) | 4 (3.1) | 7 (5.5) |  |
| Insulin | 17 (6.7) | 7 (5.5) | 10 (7.9) |  |
| GLP-1R agonist | 1 (0.4) | 1 (0.8) | 0 (0) |  |
|  |  |  |  |  |
| T2D remission^3^ | 179 (70.5) | 86 (67.7) | 93 (73.2) | - |

*Proportions of medication use and proportions in each category of glycaemic control were compared between procedures using Pearson’s chi-squared tests. Comparisons of means between procedures were performed using unpaired two-tailed t-tests.

^1^at latest postoperative follow-up, median time interval 18 months (range 12-24 months)

^2^Oral hypoglycaemic agents (metformin, sulphonylureas, thiazolidinediones)

^3^HbA1c <6.5%/48mmol/mol beyond one year postoperatively and off medications

**Supplementary Table 7. Summary of T2D remission by procedure, sex and age category showing proportions of remitters per total numbers followed-up in each category**

|  | Sex (n, %) | | Age category (yrs) (n, %) | | |
| --- | --- | --- | --- | --- | --- |
|  | **Women** | **Men** | **<40** | **40-49** | **≥50** |
| RYGBP | 68/89 (76.4) | 18/38 (47.4) | 12/18 (66.7) | 28/45 (62.2) | 46/64 (71.9) |
| SG | 55/75 (73.3) | 37/52 (71.1) | 22/24 (91.7) | 33/44 (75.0) | 37/69 (62.7) |

**Supplementary Table 8. Baseline demographic, clinical and anthropometric characteristics of completers and non-completers for each procedure**

|  | RYGBP | | | | *P** | SG | | | *P** | | |
| --- | --- | --- | --- | --- | --- | --- | --- | --- | --- | --- | --- |
|  | **Completed 1yr follow-up** | | | | | **Completed 1yr follow-up** | | | | | |
|  | **Yes** | | **No** | |  | **Yes** | **No** | | |  | |
|  |  | |  | |  |  |  | | |  | |
|  | | **n, %** | | **n, %** |  | **n, %** | | **n, %** | | |  |
| Total | | 381 (90.3) | | 41 (9.7) |  | 370 (85.4) | | 62 (14.6) | | |  |
|  | |  | |  |  |  | |  | | |  |
| Women | | 305 (80.1) | | 34 (82.9) | 0.69 | 266 (71.9) | | 44 (71.0) | | | 0.86 |
| Men | | 76 (19.9) | | 7 (17.1) |  | 104 (28.1) | | 18 (29.0) | | |  |
|  | |  | |  |  |  | |  | | |  |
| Ethnicity; white | | 302 (79.0) | | 31 (75.6) | 0.80 | 286 (77.3) | | 50 (80.6) | | | 0.38 |
| other | | 80 (21.0) | | 10 (24.4) |  | 84 (22.7) | | 12 (19.4) | | |  |
|  | |  | |  |  |  | |  | | |  |
| T2D | | 127 (33.3) | | 11 (26.8) | 0.53 | 127 (34.3) | | 20 (32.2) | | | 0.65 |
|  | |  | |  |  |  | |  | | |  |
| Hypertension | | 142 (37.2) | | 14 (34.1) | 0.72 | 156 (42.1) | | 22 (35.5) | | | 0.32 |
|  | |  | |  |  |  | |  | | |  |
| Statin therapy | | 103 (27.0) | | 11 (26.8) | 0.99 | 97 (26.2) | | 15 (24.2) | | | 0.73 |
|  | |  | |  |  |  | |  | | |  |
| OSA | | 59 (15.4) | | 4 (9.7) | 0.33 | 76 (20.5) | | 9 (14.5) | | | 0.27 |
|  | |  | |  |  |  | |  | | |  |
| Depression | | 106 (27.7) | | 16 (39.0) | 0.12 | 92 (24.9) | | 13 (21.0) | | | 0.50 |
|  | |  | |  |  |  | |  | | |  |
| Vascular disease | | 10 (2.6) | | 2 (4.9) | 0.41 | 17 (4.6) | | 0 (0) | | | 0.09 |
|  | |  | |  |  |  | |  | | |  |
| CKD | | 10 (2.6) | | 1(2.4) | 0.83 | 11(3.0) | | 1(1.6) | | | 0.93 |
|  | |  | |  |  |  | |  | | |  |
| Previous cancer | | 5 (1.3) | | 1 (2.4) | 0.56 | 6 (1.6) | | 3 (4.8) | | | 0.10 |
|  | |  | |  |  |  | |  | | |  |
|  | | **mean (SD)** | | **mean (SD)** |  | **mean (SD)** | | **mean (SD)** | | |  |
| Age (yrs) | | 43.7 (11.0) | | 43.2 (12.0) | 0.79 | 45.1 (10.2) | | 43.9 (11.2) | | | 0.39 |
|  | |  | |  |  |  | |  | | |  |
| BMI (kg/m^2^) | | 46 (5.7) | | 46.3 (6.1) | 0.77 | 50.2 (9.3) | | 49.3 (7.7) | | | 0.46 |
|  | |  | |  |  |  | |  | | |  |
| Pre-op assessment (mths) | | 7.9 (5.6) | | 8.2 (7.1) | 0.76 | 9.1 (5.8) | | 7.5 (4.4) | | | 0.04 |
|  | |  | |  |  |  | |  | | |  |
| Pre-op weight loss  (kg) | | 4.6 (6.5) | | 3.6 (7.1) | 0.52 | 5.1 (7.9) | | 4.5 (6.7) | | | 0.92 |

*Sex distribution, ethnicity distribution and prevalence of comorbidities were compared **between completers and non-completers for each procedure** using Pearson’s chi-squared tests. Comparisons of means between **between completers and non-completers** were performed **for each procedure** using unpaired two-tailed t-tests.
