# Supplementary figures and images for: Age- and sex-specific effects on weight loss outcomes in a comparison of sleeve gastrectomy and Roux-en-Y gastric bypass: a retrospective cohort study
Source: BMC Obes. 2014 Aug 11;1:12. doi: 10.1186/2052-9538-1-12 (PMC4510900; doi:10.1186/2052-9538-1-12)

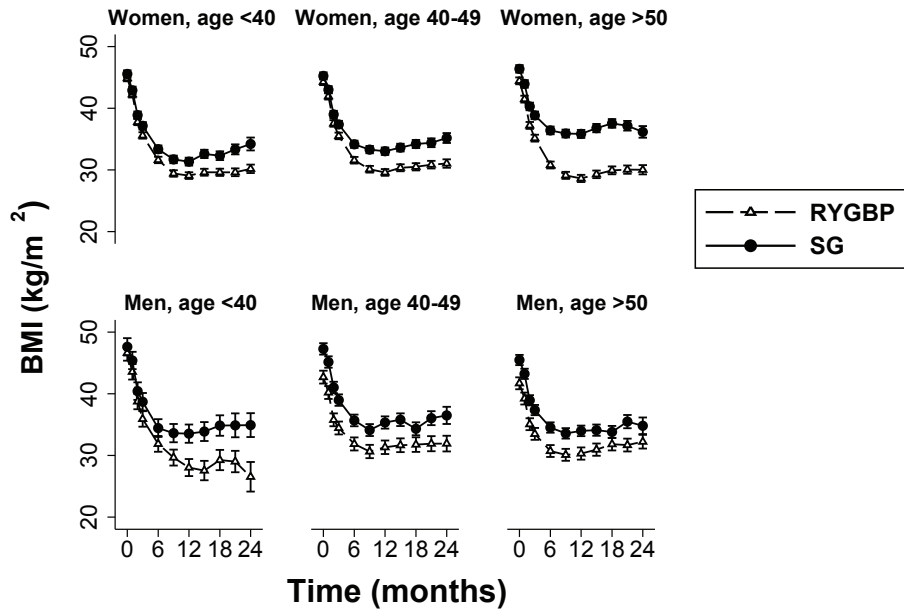

**Supplementary Figure S1**

Supplement: Supplementary file 2 — Additional file 2: Figure S1: Estimated marginal mean BMI trajectories (±SE) over a two-year postoperative period for women and men, by age categories (<40 years, 40 to 49 years, ≥50 years) in SG and RYGBP groups, excluding data from patients with a baseline BMI ≥60.0 kg/m2. (PDF 41 KB) [file 40608_2014_12_MOESM2_ESM.pdf]
